# Dexrazoxane inhibits the growth of esophageal squamous cell carcinoma by attenuating SDCBP/MDA-9/syntenin-mediated EGFR-PI3K-Akt pathway activation

**DOI:** 10.1038/s41598-024-59665-5

**Published:** 2024-04-22

**Authors:** Ruijuan Du, Nan Xiao, Li Han, KeLei Guo, Kai Li, Zhiguo Chen, Hui Zhang, Zijun Zhou, Yunlong Huang, Xulin Zhao, Hua Bian

**Affiliations:** 1 Zhang Zhongjing School of Chinese Medicine, Nanyang Institute of Technology, Nanyang, 473004 Henan People’s Republic of China; 2 Henan Key Laboratory of Zhang Zhongjing Formulae and Herbs for Immunoregulation, Nanyang Institute of Technology, No. 80, Changjiang Road, Nanyang, 473004 Henan People’s Republic of China; 3 Department of Pathophysiology, School of Basic Medical Sciences, Zhengzhou University, Zhengzhou, 450001 Henan People’s Republic of China; 4 Oncology Department, Nanyang First People’s Hospital, Nan Yang, 473004 Henan People’s Republic of China

**Keywords:** ESCC, Dexrazoxane, SDCBP/MDA-9/syntenin, EGFR, Cancer treatment, Targeted therapies, Cell growth

## Abstract

Syndecan-binding protein (SDCBP) was reported to stimulate the advancement of esophageal squamous cell carcinoma (ESCC) and could potentially be a target for ESCC treatment. There is a growing corpus of research on the anti-tumor effects of iron chelators; however, very few studies have addressed the involvement of dexrazoxane in cancer. In this study, structure-based virtual screening was employed to select drugs targeting SDCBP from the Food and Drug Administration (FDA)-approved drug databases. The sepharose 4B beads pull-down assay revealed that dexrazoxane targeted SDCBP by interacting with its PDZ1 domain. Additionally, dexrazoxane inhibited ESCC cell proliferation and anchorage-independent colony formation via SDCBP. ESCC cell apoptosis and G2 phase arrest were induced as measured by the flow cytometry assay. Subsequent research revealed that dexrazoxane attenuated the binding ability between SDCBP and EGFR in an immunoprecipitation assay. Furthermore, dexrazoxane impaired EGFR membrane localization and inactivated the EGFR/PI3K/Akt pathway. In vivo, xenograft mouse experiments indicated that dexrazoxane suppressed ESCC tumor growth. These data indicate that dexrazoxane might be established as a potential anti-cancer agent in ESCC by targeting SDCBP.

## Introduction

According to Global Cancer Statistics 2020, esophageal squamous cell carcinoma (ESCC) is the most prevalent histological variation among malignancies of the esophagus globally, ranking seventh in prevalence and sixth in mortality^1^. ESCC is one of the most aggressive types of human squamous cell carcinoma and is characterized by advanced-stage diagnosis, absence of specific targets, frequent recurrence, distant metastasis, and resistance to traditional treatment^2^. Surgical resection, chemotherapy, and radiation are the most frequently utilized treatments for ESCC patients in contemporary clinics^3^. While improvements in diagnosis and treatment approaches have helped alleviate the pain experienced by ESCC patients, the 5-year survival rate is still only 15–20%^4^. Consequently, it is crucial to understand the novel mechanisms underlying the growth of ESCC and enhance therapeutic approaches.

Syndecan binding protein (SDCBP), named melanoma differentiation-associated gene-9 (MDA-9) or Syntenin-1, is a 2100-bp gene located at 8q12 with an open reading frame of 894-bp and a roughly 33 kDa molecular mass^5,6^. Four domains, the N-terminal domain, PDZ1 domain, PDZ2 domain, and C-terminal domain, form the protein structure of SDCBP^7^. SDCBP belongs to the family of scaffold proteins due to its two PDZ domains, which mediate interactions with diverse proteins and are responsible for various signal transduction pathways in human diseases^8,9^. During cellular processes, the PDZ-2 domain of SDCBP has a higher affinity than the PDZ1 domain, and the PDZ1 domain behaves as a complementary domain. The PDZ-2 domain of SDCBP binds to C-SRC^10^, syndecan-4^11^, Frizzled 7^12^, and phosphatidylinositol 4,5-bisphosphate (PIP2)^12^. Recent studies have revealed the interaction between the PDZ1 domain of SDCBP and IGF1R^13^, Slug^14^, TGFβ1^15^, YAP1^16^, and EGFR^17^. Under normal physiological conditions, SDCBP is involved in immune response regulation^18,19^, exosome biogenesis^20,21^, cell adhesion^19,22^, and nervous system modulation^23^. During the last two decades, the SDCBP has become an increasing hotspot in cancer research. Numerous malignancies, such as melanoma, glioma, neuroblastoma, prostate, breast, and liver cancers, have increased expression of SDCBP^24^. Elevated SDCBP expression confers a tumor-supportive role by promoting cancer cell proliferation, exaggerating the process of cell migration and invasion, and facilitating tumor angiogenesis^9^. In epithelial cells, SDCBP is localized in the nucleus, cell adhesion sites, and microfilaments, colocalizing with beta-catenin, alpha-catenin, and E-cadherin at cell–cell contacts^22^. In uveal melanoma cell lines and patient tissues, SDCBP is present in nuclear and cytoplasm^25^. According to our earlier research, elevated SDCBP expression, in vitro and in vivo, accelerates ESCC progression by activating the EGFR/PI3K/Akt pathway^26^. SDCBP can bind to EGFR and sustain EGFR membrane localization, activating the EGFR/PI3K/Akt signaling pathway^26^. Accordingly, SDCBP may be a novel target for ESCC treatment.

Because of the contribution of SDCBP to tumorigenesis, researchers seek for inhibitors targeting SDCBP. Kegelman et al. reported that SDCBP is highly expressed in tissues of patients with glioma multiforme^27^. The higher the expression level of SDCBP, the poorer the prognosis of the patient and the worse the response to radiotherapy^27^. They identified a small-molecule inhibitor of SDCBP, namely PDZ1i, through a nuclear magnetic resonance (NMR) fragment-based drug design^27^. Smaller, less invasive tumors with longer lifetimes were the outcome of PDZ1i, which also made glioblastoma multiform and more radiotherapy-sensitive^27^. In another study, peptides targeting SDCBP inhibited the migration and invasion of human cancer cells with high SDCBP expression by attenuating ERK phosphorylation in the MAPK pathway^28^. The significance of SDCBP as a target for cancer treatment was demonstrated by these investigations. However, synthesizing small-molecule inhibitors is complex and expensive and is a long-term process from basic research to clinical application^29^. Furthermore, the poor stability of peptide drugs limits their applications^30^. Accordingly, exploring other ways to identify new SDCBP inhibitors is imperative.

The novel use of clinical drugs is a new strategy for developing anti-tumor drugs, which can reduce the time and cost spent on pharmacokinetic research and toxicity research^31,32^. Many drugs that have long been employed in clinics often have multiple targets, making them exhibit anti-tumor activity. For example, the hypoglycemic drug metformin and anticoagulant drug aspirin have been applied in many anti-tumor researches^33,34^. Based on the above ideas, we searched for SDCBP inhibitors by conducting a virtual screening based on the reported SDCBP structure from the database of FDA-approved drugs. Finally, the iron chelator dexrazoxane was experimentally confirmed to bind to the PDZ1 domain of SDCBP and attenuate the activity of SDCBP by disturbing complex formation between EGFR and SDCBP. Dexrazoxane—an FDA-approved iron-chelating agent—is deployed as an anti-tumor adjuvant drug to reduce anthracycline-induced myocardial toxicity^35^. One study demonstrated that a combination of metformin and dexrazoxane can inhibit the proliferation of pancreatic cancer cells^36^. Another study reported that dexrazoxane had anti-angiogenic effects in vitro and in vivo through thrombospondin-1 upregulation^37^. In this study, we identified dexrazoxane as an anti-tumor drug by targeting SDCBP. Dexrazoxane could bind to the PDZ1 domain of SDCBP and attenuate the binding ability between SDCBP and EGFR. Furthermore, dexrazoxane impaired EGFR membrane localization and inactivated the EGFR/PI3K/Akt pathway. Additionally, dexraz oxane inhibited ESCC cell proliferation and induced ESCC apoptosis and G2 phase arrest. More importantly, dexrazoxane decreased ESCC tumor growth in vivo. These data indicate that dexrazoxane is a potential anti-tumor drug by targeting SDCBP at both cellular levels and in vivo efficacy studies.

## Materials and methods

### Cell culture

HEK293T cells and ESCC cell lines (KYSE450, KYSE30, and KYSE70) with a relatively high expression of SDCBP were acquired from the cell bank of the Chinese Academy of Sciences (Shanghai, China). The normal esophageal epithelial cell line SHEE was provided by Dr. Enmin Li from the Shantou University Medical College. ESCC cells were cultivated in RPMI-1640 (Biological Industries) containing 10% fetal bovine serum (FBS), penicillin, and streptomycin. Additionally, in Dulbecco's modified Eagle's medium (Biological Industries), HEK293T cells were cultivated with 10% FBS, penicillin, and streptomycin. The cells were grown in an incubator with 5% CO_2_ humidity at 37 °C for two months. Through cellular morphology and STR analyses, the mycoplasma-free status of each cell line was confirmed.

### Plasmid construction and transfection

SDCBP and EGFR plasmids were purchased from Youbao Biotechnology Company (Changsha, China), and the sequences were verified by sequencing. SDCBP PDZ1 and PDZ2 domain-deleted mutant fragments were subcloned into pcDNA3.1–3 × Flag vector. The primers used for cloning into these constructs are as follows:

∆PDZ1-F: 5'-CGGGATCCATGACGATTACCATGCATAAG-3';

∆PDZ1-R: 5'-ATAAGAATGCGGCCGCGCAACCTCAGGAATGGTGTG-3';

∆PDZ2-F: 5'-CGGGATCCATGTCTCTCTATCCATCTCTCGAA-3';

∆PDZ2-R: 5'- ATAAGAATGCGGCCGCGCCCTGTCACGAATGGTCAT-3';

∆PDZ1 + ∆PDZ2-F: 5'- CGGGATCCATGTCTCTCTATCCATCTCTCGAA-3';

∆PDZ1 + ∆PDZ2-R: 5-ATAAGAATGCGGCCGCGCAATCCCTTGCTTAATTTC-3';

PDZ1 + PDZ2-F: 5-CGGGATCCATGCGTGAAGTCATTTTGTGT-3';

PDZ1 + PDZ2-R:5-ATAAGAATGCGGCCGCGCAACCTCAGGAATGGTGTG-3'.

According to instructions, the indicated plasmids were transfected into HEK293T cells using Simple-Fect Reagent (Signaling Dawn Biotech, Wuhan, Hubei, China).

### Computational docking model

For the structure-based virtual screening of SDCBP inhibitors, the 3D structure of SDCBP was first obtained from the Protein Data Bank^38^, and the PDB entry was 1OBZ for SDCBP^39^. AutoDock Vina, a docking software that attempts to predict the non-covalent binding of macromolecules or a small molecule (ligand) and a macromolecule (receptor), is based on known structures, or homology modeling, and so on^40^. Using AutoDock Vina, chemicals from FDA-approved drug databases were docked into the SDCBP computational model^40^. A higher anticipated protein–ligand complex was indicated by a higher negative energy score, which indicated the strength of the ligand interaction.

### MTT cell proliferation assay

KYSE450, KYSE30, and KYSE70 cells were seeded in 96-well plates and treated with varying concentrations of dexrazoxane after 24 h. The cells were then incubated with 5 mg/mL MTT solution for 4 h after dexrazoxane treatment for 24, 48, and 72 h. The medium was then discarded, and the MTT crystals were dissolved in 100 µL of DMSO. Next, cell proliferation was measured at 490 nm.

### Plate colony formation assay

First, 200 viable KYSE450, KYSE30, and KYSE70 cells were planted in triplicate onto six-well plates. The cells were treated with varying concentrations of dexrazoxane 24 h later. Following 1–2 weeks, contingent upon the cell line's growth kinetics, colonies were carefully washed with PBS and then immediately stained with 0.2% crystal violet, and images were taken under a microscope.

### Soft agar colony formation assay

Cell culture medium (1 mL) containing 2 mM glutamine, 5 μg/mL gentamycin, 0.3% soft agar, and varying concentrations of dexrazoxane, 8000 viable KYSE450, KYSE30, and KYSE70 cells were seeded in triplicate. In six-well plates, the mixture was spread out over 0.5% solidified agar in the cell culture medium. After 1–3 weeks, colonies were enumerated and captured.

### Cell apoptosis assay

After planting in 60-mm dishes, KYSE450, KYSE30, and KYSE70 cells were exposed to various dexrazoxane concentrations for 72 h. The Apoptosis Detection Kit (#640,914) was obtained from Biolegend. After two rounds of cold PBS washing, the cells were resuspended at a density of 5 × 10^6^ cells/mL. For 15 min at room temperature in the dark, the cells were treated with 5 µL of Annexin V antibody and 10 µL of propidium iodide solution. After adding 400 µL of Annexin V Binding Buffer, the cells underwent flow cytometry analysis.

### Cell cycle analysis

Following the seeding of KYSE450, KYSE30, and KYSE70 cells onto 60-mm dishes, they were exposed to varying concentrations of dexrazoxane for 48 h. Following that, cells were preserved for 24 h at − 20 °C in 70% ethanol. Propidium iodide was immediately added to the cells, followed by flow cytometry.

### Protein purification

The SDCBP protein purification was conducted as previously reported^26^. Basically, SDCBP sequence was subcloned into the pet28a vector and transformed into BL21 cells. Protein expression was then induced at 16 °C overnight with 0.5 mM IPTG. After centrifugation, cell precipitation was resuspended in lysis buffer (50 mM NaH_2_PO_4_, 300 mM NaCl, 10 mM imidazole, 1 mM phenylmethanesulfonyl fluoride, and 1% Triton X-100) and subjected to sonication. After centrifugation, the supernatants were incubated with NI–NTA agarose (QIAGEN, Germantown, MD, USA) at 4 °C for 4 h. The beads were washed four times with washing buffer (50 mM NaH_2_PO_4_, 300 mM NaCl, and 60 mM imidazole), and purified proteins were eluted with elution buffer (50 mM NaH_2_PO_4_, 300 mM NaCl, and 300 mM imidazole). The purity of SDCBP protein was determined by SDS-PAGE followed by Coomassie brilliant blue (CBB) staining.

### Pull down assay

Dexrazoxane (#S5651), trimethoprim (#S3129), zalcitabine (#S1719), emtricitabine (#S1704), lamivudine (#S1706), and nitisinone (#S5325) were acquired from Selleckchem. Drugs-conjugated Sepharose 4B beads were prepared per GE Healthcare's manufacturer's guidelines (#17-0430-01). In total, 1 mg total cell lysates or 500 ng recombinant SDCBP proteins were incubated with control Sepharose 4B beads or dexrazoxane conjugated Sepharose 4B beads in reaction buffer overnight at 4 °C. Western blotting was used to detect the presence of SDCBP protein attached to the beads after they were buffer-washed five times.

### Western blotting analysis

RIPA lysis buffer was utilized to prepare the cell lysates. The cells were then prepared by centrifugation at 4 °C, 12,000 rpm, for 15 min, following sonication. Protein concentration was determined using a BCA assay kit (#PC0020, Solarbio, Beijing, China). Subsequently, the same volumes of cell extracts were electrophoretically separated and placed onto polyvinylidene difluoride membranes. Following protein transfer, the membranes were blocked and incubated overnight at 4 °C with a specific primary antibody, followed by a 2-h incubation at room temperature with a secondary antibody. The antibodies used in this study included the following: anti-cleaved caspase-3 (Asp175) (#9664, CST); anti-cleaved caspase-7 (Asp198) (#8438, CST); anti-cleaved PARP (Asp214) (#5625, CST); anti-caspase-3 (#9662, CST); anti-caspase-7 (#9492, CST); anti-PARP (#9542, CST); anti-Bax (#5023, CST); anti-SDCBP (#H00006386-M01, Abnova Corporation); anti-p-EGFR (Tyr1068) (#3777, CST); anti-p-PI3K p85α (Tyr467) (#sc-293115, Santa Cruz); anti-EGFR (#4267, CST); anti-PI3K p85 (#4257, CST); anti-Akt(pan) (#4691, CST); anti-p-AKT(Ser473) (#4060, CST); anti-Flag (#F1804, Sigma); anti-HA (#3724, CST); anti-GAPDH (#HRP-60004, Proteintech). An enhanced chemiluminescence reagent (#34579, ThermoFisher Scientific) was employed to visualize the protein bands.

### SDCBP knockdown using shRNAs and virus infection

The shRNAs for SDCBP were constructed as previously reported^26^. Next, 4 μg of pMD2.G, 4 μg of psPAX2 packaging plasmids, and 4 μg of pLKO.1 were added. A single plasmid containing the designated shRNAs was transfected into HEK293T cells. The virus particles were gathered and filtered using a 0.45-mm filter two days after transfection. To produce stable KYSE450 cell lines with SDCBP knockdown, the lentivirus was combined with 2 mg/mL puromycin for selection.

### Immunoprecipitation

Cell lysates from KYSE450, KYSE30, and HEK293T cells, which were treated with 0, 25, and 50 μM dexrazoxane for 24 h, were generated by centrifuging the samples for 15 min at 4 °C at 12,000 rpm, after which they were cleaned using lysis buffer for 30 min at 4 °C. Following an overnight incubation period at 4 °C with anti-EGFR (1:100, #4267, CST) for ESCC cells and anti-Flag (1:100, #F1804, Sigma) and anti-HA (1:100, #3724, CST) for HEK293T cells, cell lysates were incubated for 3 h at 4 ℃ with protein A/G agarose beads (#sc-2003, Santa Cruz). After washing the conjugated beads with lysis buffer and twice with PBS, the immune complexes were eluted for 5 min at 95 °C using 1 × sample loading buffer. Following immunoprecipitation, proteins were quantified using Western blotting.

### Immunofluorescence

KYSE30 and KYSE450 cells were seeded on glass slides. After 24 h, the cells were treated with 50 μM dexrazoxane for 8 h. Following a PBS wash, the cells were fixed for 30 min with 4% paraformaldehyde at room temperature and then permeabilized for 10 min on ice using 0.2% Triton X-100 solution. Following an overnight staining period at 4 °C with anti-EGFR (diluted in 2% bovine serum albumin and 5% goat serum), the cells were stained with the secondary antibody (diluted in PBS). Finally, cell nuclei were stained with DAPI for 15 min at room temperature.

### Cell-derived xenograft mouse model

The animal experiments in this study were approved by the Ethics Committee of Nanyang Institute of Technology (approval no. NYISTIRB-2021-006). All experiments followed the institutional guidelines and regulations, and the authors complied with the ARRIVE guidelines. Six- to eight-week-old female NU/NU mice were used for animal experiments. KYSE450 cells (1 × 10^7^ cells/mouse) were subcutaneously injected into the right flank of each mouse. After one week, when tumors reached an average volume of approximately 60 mm^3^, mice were divided into three groups for further experiments as follows: (1) vehicle-treated group (n = 7), (2) group treated with 10 mg/kg of dexrazoxane (n = 7), and (3) group treated with 30 mg/kg of dexrazoxane (n = 7). Dexrazoxane was dissolved in a 0.167 mol/L sodium lactate solution, and the vehicle-treated group was injected with 0.167 mol/L sodium lactate. Dexrazoxane was administered by intraperitoneal injection every two days. Tumor size and body weight were measured every four days. The tumor volume (mm^3^) was determined as follows: (length × width × height × 0.52). After 25 days of dexrazoxane treatment, the mice were euthanized, and tumors were extracted.

### Immunohistochemistry

For the analysis, tumor tissues from the CDX model were embedded in paraffin. Briefly, every specimen was sectioned into 4 μm slides after being deparaffinized and rehydrated. The samples were then boiled in sodium citrate buffer (10 mmol/L, pH 6.0) for 10 min for antigen retrieval. After that, the samples were exposed to 3% H_2_O_2_ for 10 min. Next, 10% goat serum albumin was added to each slide and incubated at room temperature for 1 h in a humidified environment. Then, the slides were left overnight at 4 °C to be treated with primary antibodies. The following day, the slides were incubated with a secondary antibody for 30 min at room temperature. The sections were finally counterstained with hematoxylin, dehydrated, covered, and visualized. For each section, ten random images were collected, and the positivity rate was measured using Image-Pro Plus software (v.6.0).

### Statistical analysis

Statistical analyses were performed using GraphPad Prism version 6. All quantitative results are expressed as mean values ± S.D. Significant differences between the two groups were compared using the Student^’^s t-test. Statistical significance was set at a p-value of < 0.05.

## Results

### Dexrazoxane targets the PDZ1 domain of SDCBP

**Figure 1:**
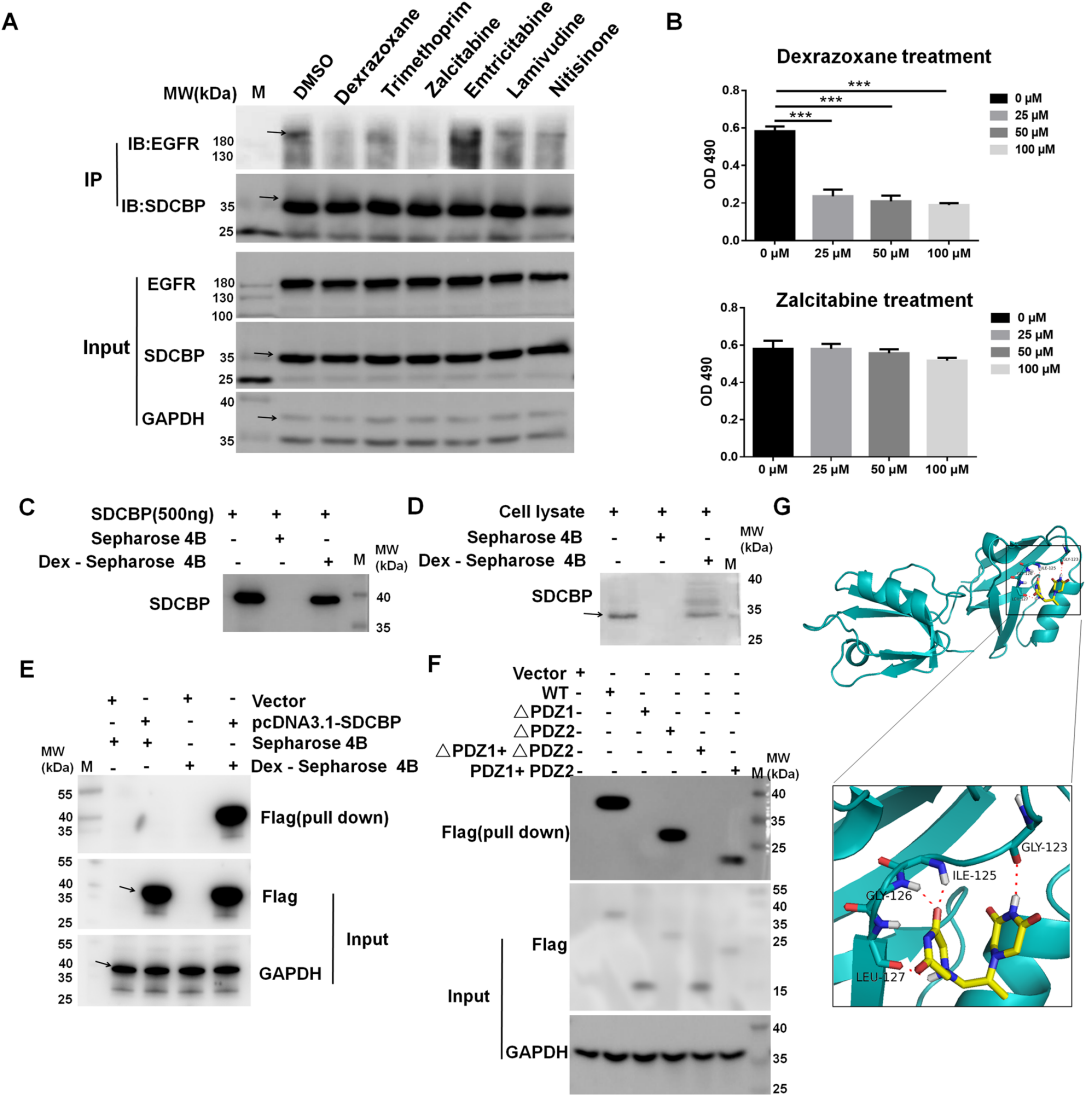
Dexrazoxane targets the PDZ1 domain of SDCBP. (**A**) KYSE450 cells were treated with the indicated drugs at a concentration of 100 μM. Lysates from the cells were immunoprecipitated with anti-SDCBP and then immunoblotted with the indicated antibodies. (**B**) KYSE450 cells were treated with different concentrations of dexrazoxane or zalcitabine. Cell proliferation was measured at 72 h by MTT assay. The binding of dexrazoxane to recombinant SDCBP protein (**C**) or SDCBP in KYSE450 cell lysates (**D**) was determined using Sepharose 4B or dexrazoxane-conjugated Sepharose 4B beads. (**E**) A vector or pcDNA3.1-SDCBP plasmid was transfected into HEK293T cells. The binding of dexrazoxane to exogenous SDCBP was determined using Sepharose 4B or dexrazoxane-conjugated Sepharose 4B beads. (**F**) The flag-tagged SDCBP deletion mutants were transfected into HEK293T cells. The binding of dexrazoxane to exogenous different SDCBP fragments was determined using dexrazoxane-conjugated Sepharose 4B beads. (**G**) Model of dexrazoxane binding with SDCBP. Upper: dexrazoxane binding with the PDZ1 domain of SDCBP; lower: enlarged view of the binding. SDCBP was shown as a ribbon representation, and dexrazoxane was shown as a stick representation. Cropped images and uncropped blots are displayed in Supplementary Figs. S1 and S2.

Structure-based virtual screening was performed using compounds from FDA-approved drug databases based on the protein structure of the SDCBP. The drugs were ranked by their negative energy values, representing the binding affinity between them and SDCBP. We focused on the top ten drugs illustrated in Table 1, among which glutethimide and methyprylon belong to the hypnotic and sedative agents. Additionally, gemcitabine is a known anti-cancer agent, and enprofylline has cardiac and neurological toxicity. Therefore, these four drugs were excluded, and the remaining six drugs were zalcitabine, emtricitabine, lamivudine, dexrazoxane, nitisinone, and trimethoprim. We then determined whether these drugs could influence the binding ability between SDCBP and EGFR, which reflects the activity of SDCBP. Figure 1A depicts that at 100 μM, zalcitabine and dexrazoxane eliminated the binding between SDCBP and EGFR. We then detected the antiproliferative effect of zalcitabine and dexrazoxane in KYSE450 cells and observed that zalcitabine did not inhibit cell proliferation, while 25 μM dexrazoxane significantly decreased cell viability (Fig. 1B). Herein, dexrazoxane was considered for further confirmation as a drug both targeting SDCBP and possessing an anti-cancer effect.

**Table 1:** Top 10 FDA approved drug list according to the binding affinity.

Drug name	Structure	Binding energy (kcal/mol)
Zalcitabine		− 8.476
Emtricitabine		− 7.694
Lamivudine		− 7.573
Dexrazoxane		− 7.285
Gemcitabine		− 7.007
Glutethimide		− 6.605
Methyprylon		− 6.412
Enprofylline		− 6.405
Nitisinone		− 6.398
Trimethoprim		− 6.376

We then used dexrazoxane-conjugated Sepharose 4B beads to verify whether dexrazoxane targeted the SDCBP. The results indicated that dexrazoxane could directly bind to recombinant SDCBP protein **(**Fig. 1C), SDCBP protein in KYSE450 cell lysates (Fig. 1D), and exogenous SDCBP protein in HEK293T cells (Fig. 1E). To further determine whether dexrazoxane targeted the PDZ1 or PDZ2 domain of SDCBP, we transfected several vectors with different SDCBP PDZ domain deletions into HEK293T cells. Dexrazoxane-conjugated Sepharose 4B beads were used to pull down the cell lysates, and Fig. 1F displayed that dexrazoxane interacted with the PDZ1 domain of the SDCBP. The docking results showed that hydrogen bonds were formed between dexrazoxane and SDCBP at the Gly123, Ile125, Gly126, and Leu127 amino acid sites (Fig. 1G). These data suggest that dexrazoxane binds to the PDZ1 domain of the SDCBP.

### Dexrazoxane inhibits ESCC cell proliferation through SDCBP

**Figure 2:**
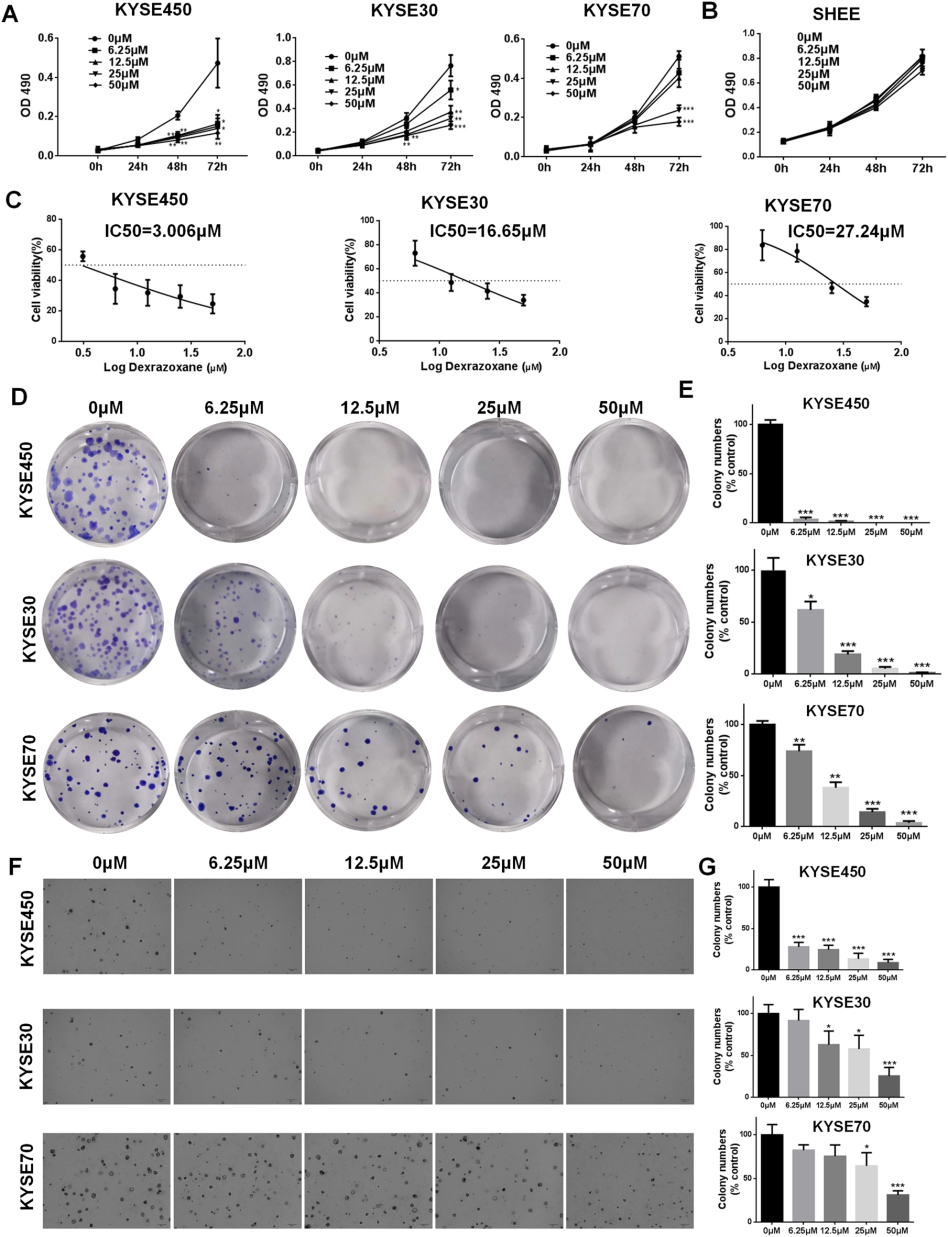
Dexrazoxane inhibits ESCC cell growth. The effect of dexrazoxane on the cell proliferation of KYSE450, KYSE30, and KYSE70 cells (**A**) and SHEE cell (**B**) was detected by the MTT assay. (**C**) KYSE450, KYSE30, and KYSE70 cells were treated with different concentrations of dexrazoxane for 72 h. The cell viability was assessed by MTT assay, and IC_50_ values were calculated. (**D**,**E**) Plate colony formation assay was performed after administration of different concentrations of dexrazoxane. (**F**,**G**) The effect of dexrazoxane on the anchorage-independent colony growth of KYSE450, KYSE30, and KYSE70 cells was evaluated. *P < 0.05, **P < 0.01, ***P < 0.001. Error bars represent the mean ± SD of at least three independent experiments.

We then used MTT and plate colony formation assays to measure the proliferation of KYSE450, KYSE30, and KYSE70 cells following treatment with different concentrations of dexrazoxane. The findings showed that ESCC cell growth was dose-dependently reduced by dexrazoxane (Fig. 2A). The effect of dexrazoxane on cell proliferation was also evaluated in the normal esophageal epithelial cell line, SHEE, and the results revealed that 50 μM dexrazoxane did not significantly inhibit SHEE cell growth (Fig. 2B). The calculated IC_50_ of dexrazoxane was 3.006 μM for KYSE450 cells, 16.65 μM for KYSE30 cells, and 27.24 μM for KYSE70 cells (Fig. 2C). Besides, plate colony formation assay showed that dexrazoxane strongly suppressed colony formation by KYSE450, KYSE30, and KYSE70 cells (Fig. 2D,E). In an anchorage-independent cell growth assay, dexrazoxane showed inhibitory effects on the colony formation of KYSE450, KYSE30, and KYSE70 cells in agarose (Fig. 2F,G).

**Figure 3:**
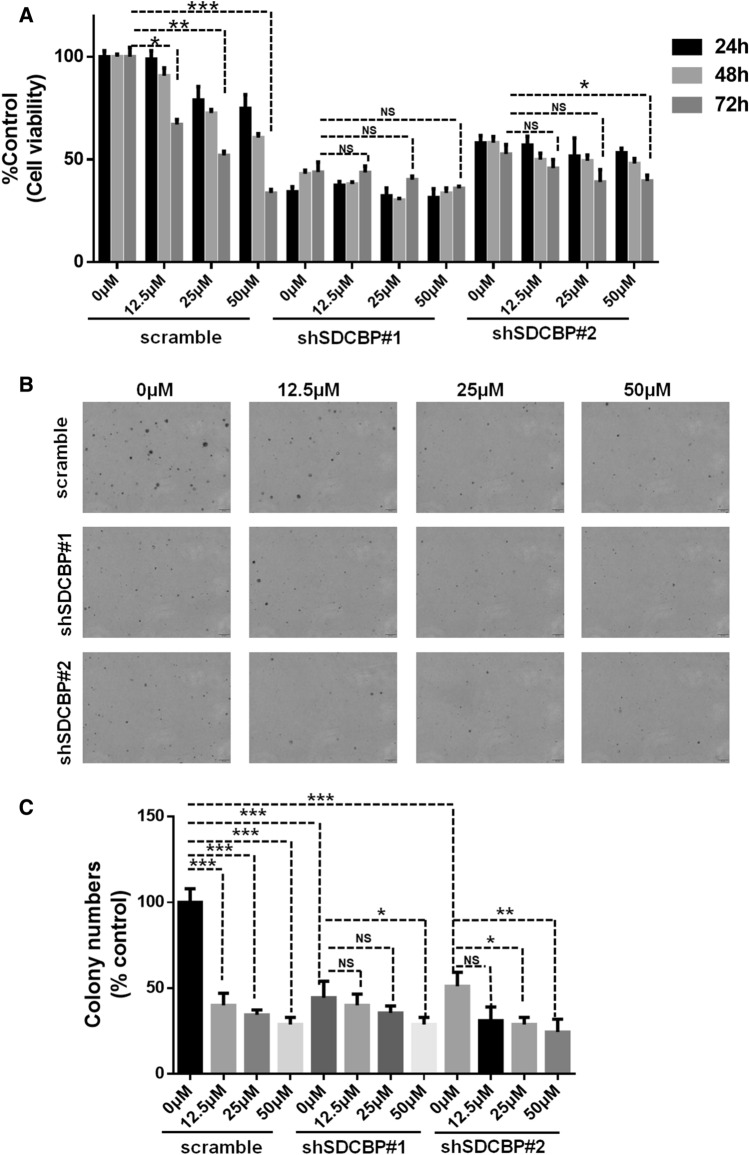
Dexrazoxane inhibits ESCC cell growth through SDCBP. (**A**) The effect of dexrazoxane treatment on cell growth was assessed in KYSE30 scramble cells or SDCBP knockdown cells. (**B**,**C**) The effect of dexrazoxane treatment on the anchorage-independent colony formation in KYSE30 scramble cells or SDCBP knockdown cells. *P < 0.05, **P < 0.01, ***P < 0.001. Error bars represent the mean ± SD of at least three independent experiments.

Furthermore, we verified whether dexrazoxane inhibits ESCC cell proliferation by targeting SDCBP. After SDCBP knockdown in KYSE30 cells, treatment with dexrazoxane had almost no effect on cell proliferation and anchorage-independent colony formation compared with control cells (Fig. 3A–C). These data indicate that dexrazoxane inhibits ESCC cell proliferation via SDCBP.

### Dexrazoxane induces ESCC cell apoptosis and cell cycle arrest at the G2 phase

**Figure 4:**
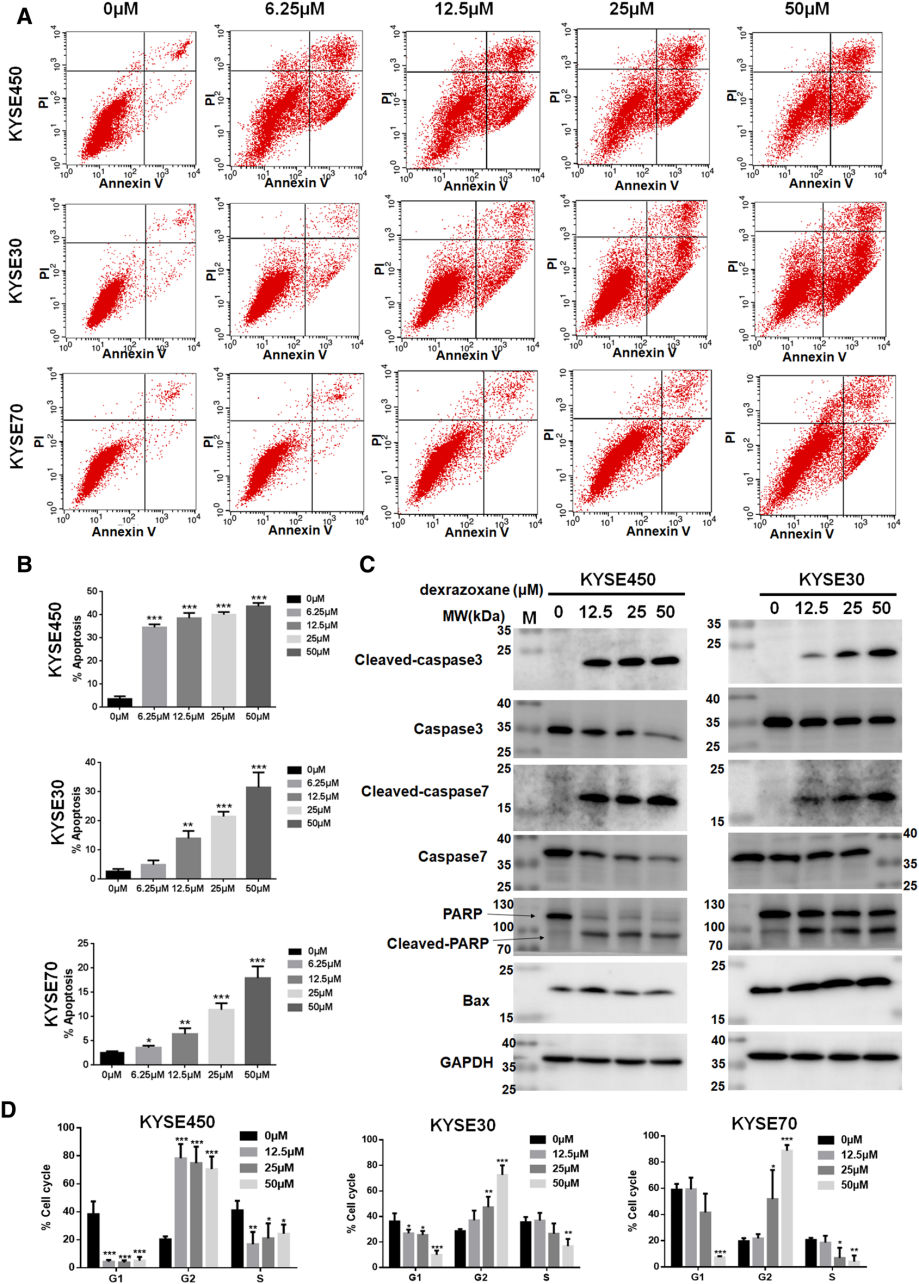
Dexrazoxane induces ESCC cell apoptosis and cell cycle arrest at the G2 phase. (**A**) The representative images of cell apoptosis after dexrazoxane treatment at 72 h in KYSE450, KYSE30, and KYSE70 cells were shown. (**B**) Cell apoptosis at 72 h was analyzed based on annexinV + gating. (**C**) Apoptotic protein markers were measured after dexrazoxane treatment at 72 h in KYSE450 and KYSE30 cells. (**D**) The effect of dexrazoxane treatment on the cell cycle at 48 h was statistically analyzed. *P < 0.05, **P < 0.01, ***P < 0.001. Error bars represent the mean ± SD of at least three independent experiments. Cropped images and uncropped blots are displayed in Supplementary Fig. S3.

Cell apoptosis and the cell cycle were measured after dexrazoxane treatment. Figure 4A,B present significant induction of apoptosis when the dexrazoxane concentration was 12.5 μM in KYSE450, KYSE30, and KYSE70 cells. We next used Western blotting to investigate the expression of apoptosis biomarkers to further evaluate the effects of dexrazoxane on apoptosis after dexrazoxane treatment. We observed increased expression of cleaved caspase-3, cleaved caspase-7, cleaved PARP, and Bax (Fig. 4C). Furthermore, dexrazoxane markedly induced cell cycle arrest in the G2 phase in KYSE450, KYSE30, and KYSE70 cells (Fig. 4D).

### Dexrazoxane decreases SDCBP activity and downstream signaling

**Figure 5:**
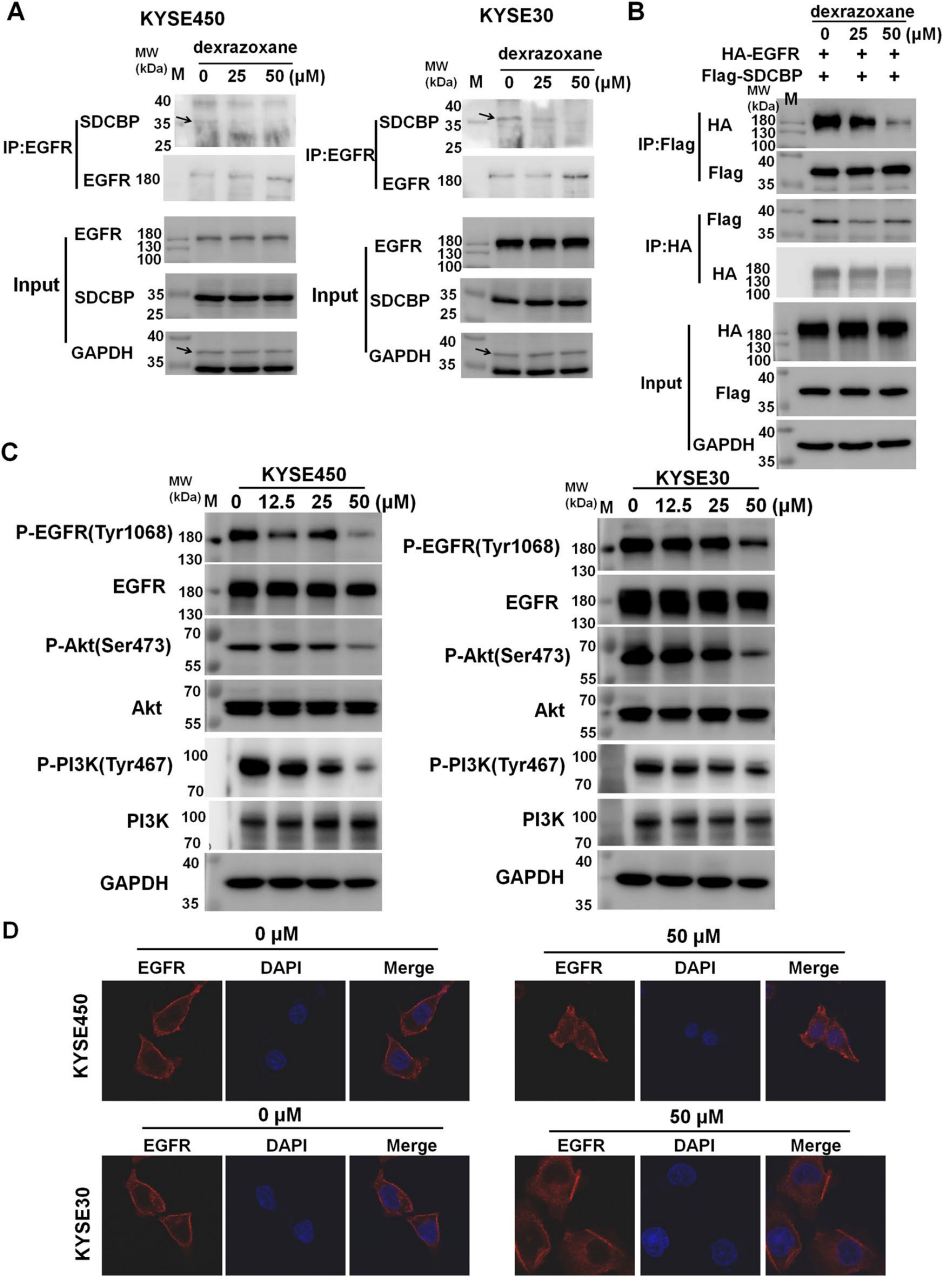
Dexrazoxane decreases SDCBP activity and downstream signaling. (**A**) KYSE450 and KYSE30 cells were treated with different concentrations of dexrazoxane. Lysates from KYSE450 and KYSE30 cells were immunoprecipitated with anti-EGFR and then immunoblotted with the indicated antibodies. (**B**) After transfecting HEK293T cells with EGFR and overexpressing SDCBP the plasmid, the lysates were immunoprecipitated using either anti-Flag or anti-HA and subsequently subjected to immunoblotting using the designated antibodies. (**C**) KYSE450 and KYSE30 cells were treated with different concentrations of dexrazoxane. Lysates were subjected to Western blotting to detect EGFR/PI3K/Akt pathway proteins. (**D**) KYSE450 and KYSE30 cells were treated with dexrazoxane, and EGFR localization was evaluated through an immunofluorescence assay. Cropped images and uncropped blots are displayed in Supplementary Figs. S4–S6.

SDCBP was reported to promote ESCC progression by interacting with EGFR and activating the EGFR/PI3K/Akt signaling pathway^26^. We then explored whether dexrazoxane interfered with the interaction between SDCBP and EGFR and inhibited the EGFR/PI3K/Akt pathway. Immunoprecipitation assays demonstrated that in KYSE450 and KYSE30 cells, dexrazoxane inhibited the binding ability between EGFR and SDCBP (Fig. 5A). To confirm these data, we transfected SDCBP and EGFR into HEK293T cells. The results demonstrated that dexrazoxane prevented the interaction between exogenous SDCBP and EGFR (Fig. 5B). Then, the effect of dexrazoxane on the activity of EGFR/PI3K/Akt pathway was evaluated. We observed that dexrazoxane decreased the expression of p-EGFR, p-PI3K, and p-Akt in KYSE450 and KYSE30 cells (Fig. 5C). EGFR membrane localization was responsible for EGFR/PI3K/Akt pathway activation and was maintained by SDCBP^26^. We then measured the effect of dexrazoxane on EGFR membrane localization. Figure 5D indicates that the internalization of EGFR from the cell membrane into the cytoplasm was facilitated by the administration of dexrazoxane. Consequently, dexrazoxane attenuated the binding between SDCBP and EGFR, impaired EGFR membrane localization, and inactivated the EGFR/PI3K/Akt pathway.

### Dexrazoxane inhibits ESCC tumor growth in vivo

**Figure 6:**
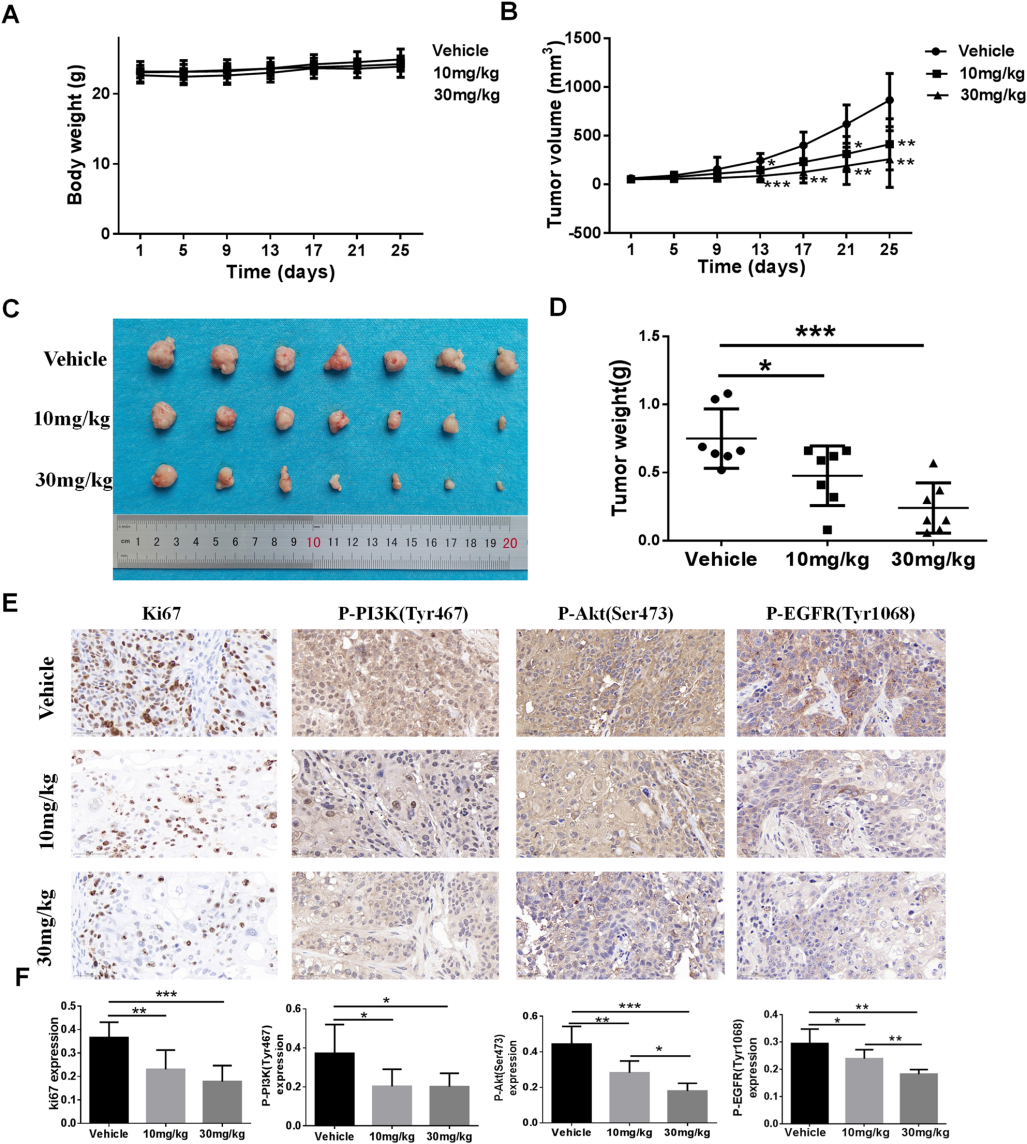
Dexrazoxane inhibits ESCC tumor growth in vivo. (**A**) Mice body weight and tumor volume (**B**) from vehicle-treated or dexrazoxane-treated groups were measured every four days. (**C**) At the end of the trial, mice with tumors were removed. Images of the tumors in each group were displayed. (**D**) Following tumor excision, tumor weight was determined. (**E**,**F**) Immunohistochemistry was used to evaluate Ki67, P-EGFR, P-PI3K, and P-Akt in xenograft tissues that had been extracted. *P < 0.05, **P < 0.01, ***P < 0.001. Error bars represent the mean ± SD.

We further assessed the anti-tumor activity of dexrazoxane in vivo based on prior evidence. Mice were administered intraperitoneal injections of vehicle or dexrazoxane every other day for 25 days. The mice were weighed to assess the possible toxicity of dexrazoxane. The results indicated that 10 and 30 mg/kg dexrazoxane did not significantly affect mice's body weight (Fig. 6A). Compared to vehicle-treated group, the injection of dexrazoxane dramatically suppressed tumor volume and weight (Fig. 6B–D). Furthermore, the inhibition of dexrazoxane on tumor cell proliferation was confirmed by IHC staining of ki67, and we observed decreased ki67 expression after dexrazoxane treatment (Fig. 6E,F). Finally, the effect of dexrazoxane on p-EGFR, p-PI3K and p-Akt expression was examined by IHC. The results revealed that p-EGFR, p-PI3K and p-Akt levels were reduced after dexrazoxane administration (Fig. 6E,F). Accordingly, dexrazoxane inhibited ESCC tumor growth and PI3K/Akt activation in vivo.

## Discussion

ESCC is a malignant tumor and a public health issue worldwide. For most patients diagnosed with ESCC, the traditional and most frequent treatment methods are surgery, chemotherapy, and radiotherapy, which will cause resistance, high recurrence rates, and a series of dose-limiting toxicities^3,41^. Due to the high tumor heterogeneity of ESCC, it is crucial to identify efficient molecular targets and investigate their inhibitors^42^. Although multiple molecular targets have been investigated in ESCC, few targeted therapies have been recommended for ESCC treatment in clinic^41,43^. Until now, EGFR, HER2, and VEGF have been the main targets used in clinical trials, and their efficiency is limited^41,43^. Other treatments, including immune checkpoint inhibitors, tumor vaccines, and epigenetic-targeted therapy, remain in progress^41,43^. Furthermore, in preclinical and clinical studies, using natural substrates isolated from medicinal plants has obtained promising outcomes for ESCC^44–47^; however, further assessment of the clinical application is time-consuming and is still ongoing. The application of FDA-approved drugs for cancer treatment has been a hotspot for studying pharmacokinetics, establishing safety in humans, and evaluating toxicity^31,32^. Consequently, it may be an intriguing strategy to utilize FDA-approved drugs for ESCC treatment, and several drugs have been demonstrated to have anti-cancer effect in ESCC, such as dasabuvir^48^ (for hepatitis C virus treatment), azelnidipine^49^ (for hypertension), penfluridol^50^ (an antipsychotic drug), and antitussive^51^ (an antitussive agent). In this study, we identified an FDA-approved drug, dexrazoxane, as a potential anti-cancer drug for ESCC both in vitro and in vivo.

Dexrazoxane, a cardioprotective agent, is used for female patients with metastatic breast cancer who have received doxorubicin treatment with a cumulative dose of 300 mg/m^2^, and doctors believe that continuing to use doxorubicin is beneficial^35^. To date, few studies have reported the anti-tumor potential of dexrazoxane. Maloney et al. reported that dexrazoxane administration at low repeated doses could largely inhibit angiogenesis in vitro and in vivo^37^. Dexrazoxane suppressed the proliferation of human umbilical vein endothelial cells (HUVECs) and human dermal microvascular endothelial cells (HDMECs), with IC_50_ values of 71 μM for HUVECs and 52 μM for HDMECs^37^. Our study showed no significant proliferation inhibition of 50 μM dexrazoxane on normal esophagus epithelial cells. Dexrazoxane was also reported to trigger the accumulation of HUVECs and HDMECs in the G2 phase, which is consistent with our data that dexrazoxane induced ESCC cell cycle arrest at the G2 phase^37^. Although dexrazoxane inhibits the growth of vascular endothelial cells, there are no reported side effects of vascular injury by dexrazoxane^35,37^. According to Maloney et al., dexrazoxane did not influence VEGF-stimulated cell migration^37^. Further studies are required to investigate whether dexrazoxane affects ESCC cell migration and metastasis. Another study showed that 50–100 μM dexrazoxane alone slightly inhibited cell growth; however, cell proliferation was strongly suppressed when combined with metformin^36^. This may indicate that a synergistic anti-tumor effect may be obtained using the reported FDA-approved drugs. Inflammation and oxidative stress are critical contributors to cancer progression^52,53^, and anti-inflammatory or antioxidant therapies have the potential to be effective in cancer treatment^54,55^. Dexrazoxane has been reported to exert anti-inflammatory and antioxidant effects by inhibiting oxidative stress and endoplasmic reticulum stress, and suppressing systemic inflammation in Parkinson's disease^56^. Therefore, the mechanism of action of dexrazoxane can extend to its anti-inflammatory and antioxidant roles in future studies.

Initially, SDCBP was found to be an upregulated protein during terminal differentiation in human malignant melanoma by interferon and mezerein^5,57^. In the past 20 years, numerous significant biological processes in malignancies, including angiogenesis, autophagy, chemoresistance, cell invasion and metastasis, and cell proliferation, have been linked to SDCBP^58^. Scaffold proteins mediate various biological functions by interacting with various partners. It has been demonstrated that SDCBP binds to various partners, triggering the activation of the downstream Wnt-β-catenin pathway, p38 MAPK, NF-κB, STAT3, and PI3K-Akt signaling in malignancies^10,12,17,59^. Notably, SDCBP interacts with EGFR in urothelial cell carcinoma and modulates EGFR signaling^17^. Another study showed that pEGFR (Tyr-1068) expression significantly decreased after SDCBP knockdown in glioma cells and that EGFR signaling activation was necessary for SDCBP–mediated protective autophagy^60^. Consistent with the published data on the relationship between SDCBP and EGFR, in our previous study, SDCBP was abundantly expressed in ESCC and accelerated the formation of ESCC tumors by interacting with EGFR and initiating the EGFR-PI3K-Akt signaling pathway^26^. EGFR was found to be overexpressed and amplified in ESCC^61,62^, and EGFR overexpression was associated with lymph node metastasis, disease-free survival, and overall survival^61^. EGFR-targeted therapies such as cetuximab and bevacizumab have been used in clinical trials to treat ESCC, and some have obtained promising results^43,63^. The PI3K-AKT pathway was the downstream effector of EGFR, and the core components of the PI3K-AKT pathway were found to be altered and activated^64,65^. Therefore, targeting the PI3K-AKT pathway is also an interesting field, and inhibitors of the PI3K-AKT pathway have been on the way in preclinical or clinical attempts^66,67^. We have previously indicated that SDCBP could promote ESCC tumor growth by activating the EGFR-PI3K-Akt pathway^26^. Thus, we hypothesized that inhibitors targeting SDCBP may attenuate the activation of the EGFR-PI3K-Akt pathway and consequently suppress ESCC progression. In this study, we screened dexrazoxane as a potential SDCBP inhibitor, and dexrazoxane was confirmed to attenuate EGFR-PI3K-Akt pathway activation. Dexrazoxane may imitate the anti-tumor effects caused by EGFR and PI3K-Akt inhibitors. The present study found that dexrazoxane inhibited ESCC cell proliferation and induced apoptosis and cell cycle arrest at the G2 phase. Furthermore, dexrazoxane decreased ESCC tumor growth in vivo. Further mechanistic studies demonstrated that dexrazoxane targeted the PDZ1 domain of SDCBP and interfered with the downstream EGFR/PI3K/Akt pathways. This study offers a comprehensive understanding of the anti-tumor effects of dexrazoxane in ESCC.

## Conclusions

**Figure 7:**
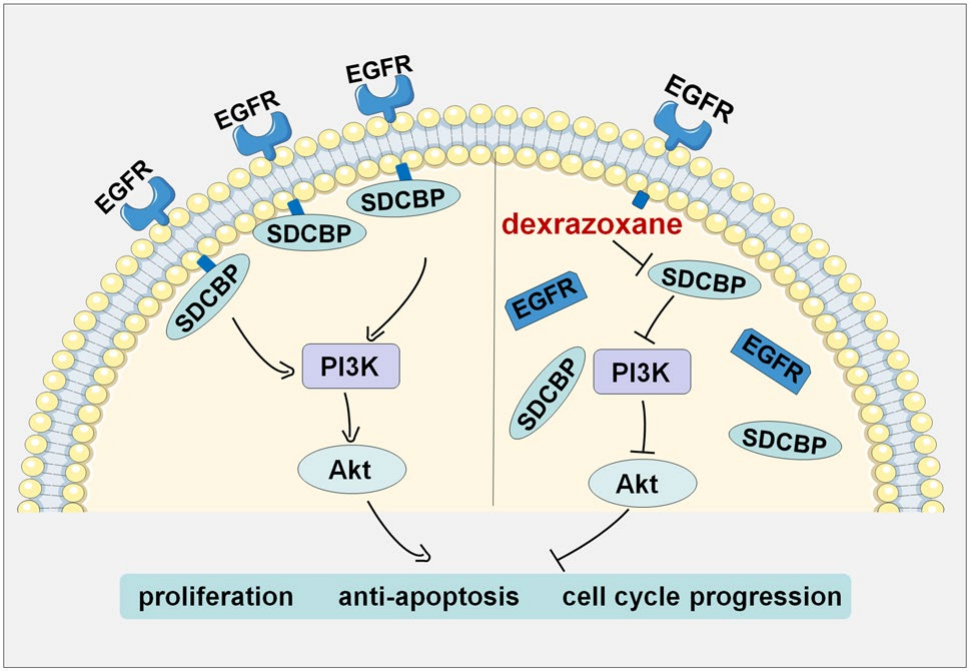
A proposed scheme illustrating the effect and mechanism of dexrazoxane on ESCC progression. Dexrazoxane targeted PDZ1 domain of SDCBP and attenuated the binding ability between SDCBP and EGFR. Moreover, dexrazoxane impaired EGFR membrane localization and inhibited EGFR/PI3K/Akt pathway. Finally, ESCC progression was inhibited due to decreased cell proliferation, increased cell apoptosis, and cell cycle arrest.

In the present study, as presented in Fig. 7, we found that dexrazoxane could bind to the PDZ1 domain of SDCBP and attenuate the binding between SDCBP and EGFR. Mechanistic studies have demonstrated that dexrazoxane impairs EGFR membrane localization and inhibits the EGFR/PI3K/Akt pathway. Functionally, dexrazoxane inhibited ESCC cell proliferation and induced apoptosis and G2 phase arrest. More importantly, dexrazoxane decreased ESCC tumor growth in vivo. Overall, our data demonstrated that dexrazoxane may be a potential anti-tumor drug for ESCC by targeting SDCBP.

### Supplementary Information

Supplementary Figures.

## Data Availability

The datasets used and/or analyzed during the current study are available from the corresponding author upon reasonable request.

## Abbreviations

ESCC: Esophageal squamous cell carcinoma
SDCBP: Syndecan binding protein
MDA-9: Melanoma differentiation associated gene-9
FDA: Food and drug administration
