# Supplementary material for: Dexrazoxane inhibits the growth of esophageal squamous cell carcinoma by attenuating SDCBP/MDA-9/syntenin-mediated EGFR-PI3K-Akt pathway activation
Source: Sci Rep. 2024 Apr 22;14:9167. doi: 10.1038/s41598-024-59665-5 (PMC11035576; doi:10.1038/s41598-024-59665-5)
Supplement: Supplementary file 1 — Supplementary Figures. [file 41598_2024_59665_MOESM1_ESM.pdf]

Figure S1

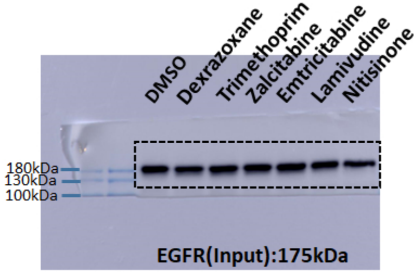

Original image of Figure 1A
with colorful protein ladder.

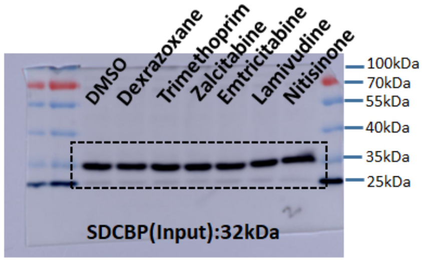

Original image of Figure 1A
with colorful protein ladder.

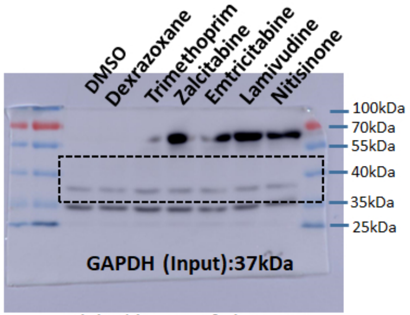

Original image of Figure 1A
with colorful protein ladder.

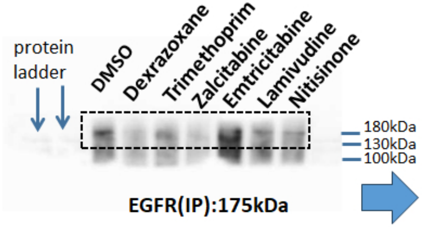

Original image of Figure 1A
with light gray protein ladder.

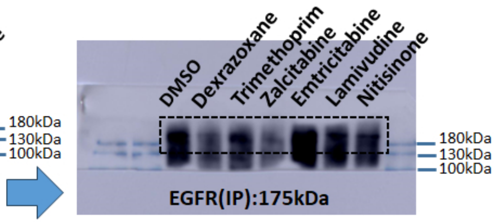

Original image of Figure 1A
with colorful protein ladder.

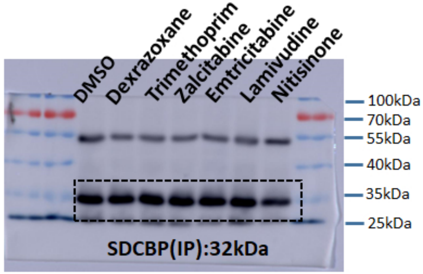

Original image of Figure 1A
with colorful protein ladder.

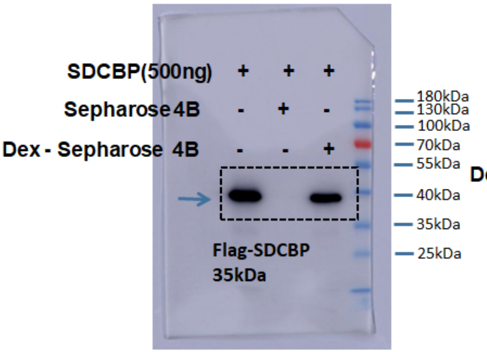

Original image of Figure 1C
with colorful protein ladder.

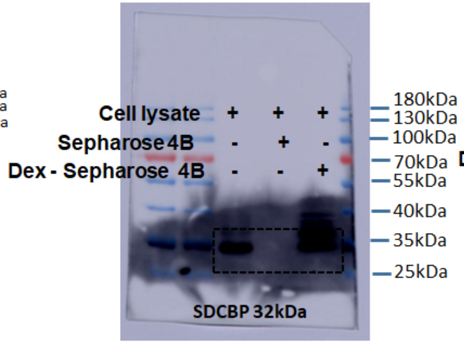

Original image of Figure 1D
with colorful protein ladder.

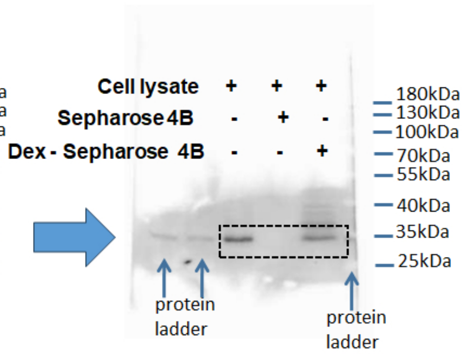

Original image of Figure 1D with
gray protein ladder.

Figure S2

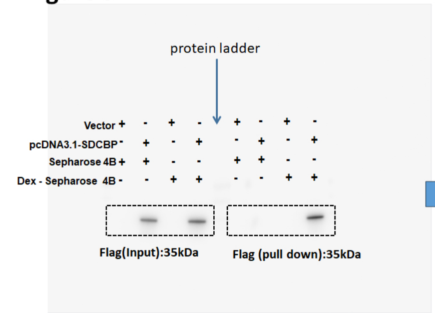

Original image of Figure 1E with shorter exposure time and lighter gray protein ladder .

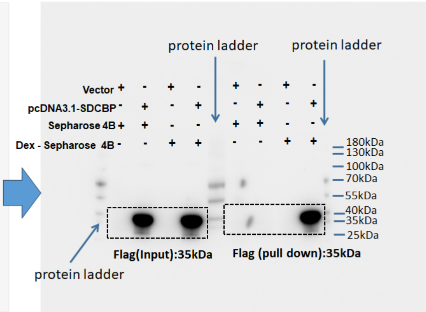

Original image Figure 1E with longer exposure time and obvious gray protein ladder.

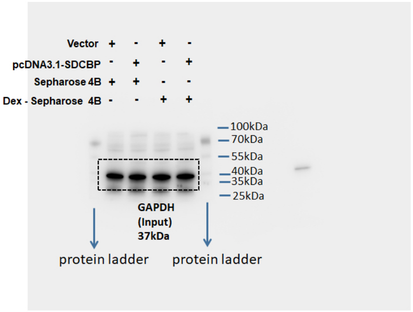

Original image Figure 1E with gray protein ladder.

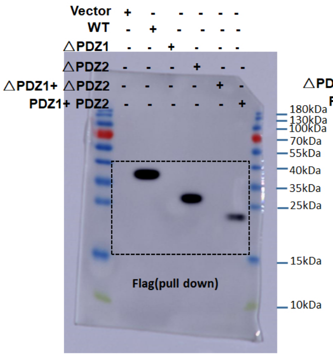

Original image of Figure 1F with colorful protein ladder.

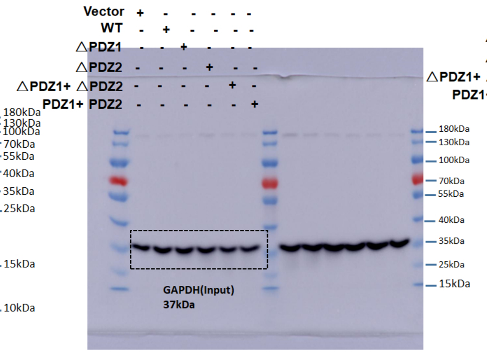

Original image of Figure 1F with colorful protein ladder.

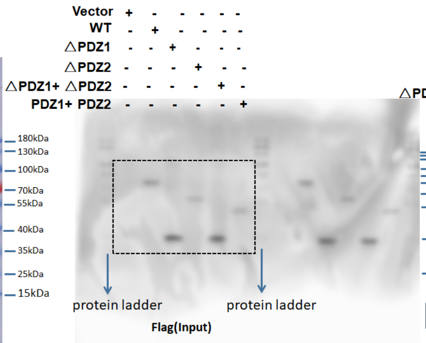

Original image of Figure 1F with gray protein ladder.

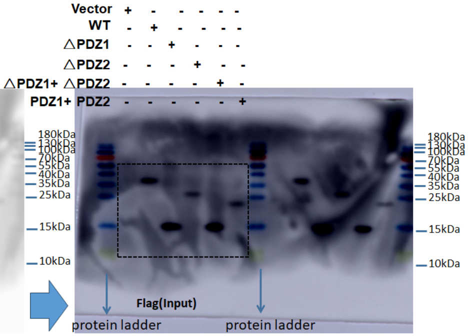

Original image of Figure 1F with colorful protein ladder.

Figure S3

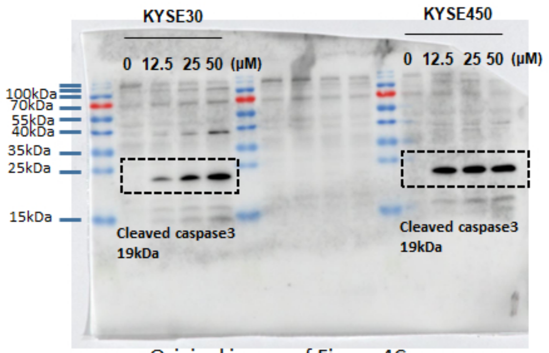

Original image of Figure 4C
with colorful protein ladder.

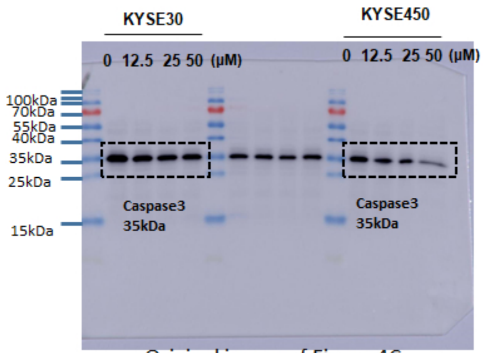

Original image of Figure 4C
with colorful protein ladder.

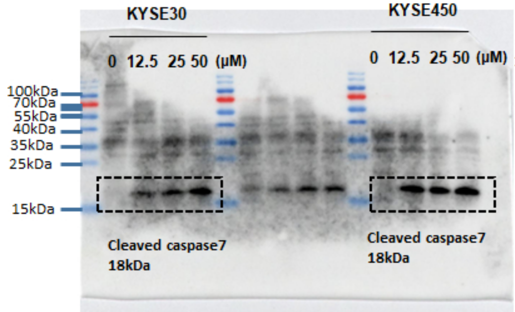

Original image of Figure 4C
with colorful protein ladder.

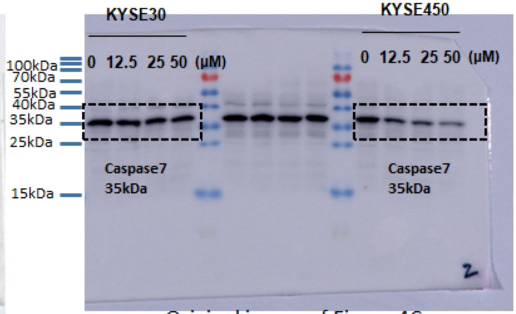

Original image of Figure 4C
with colorful protein ladder.

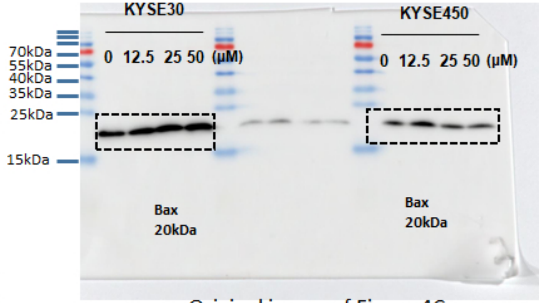

Original image of Figure 4C
with colorful protein ladder.

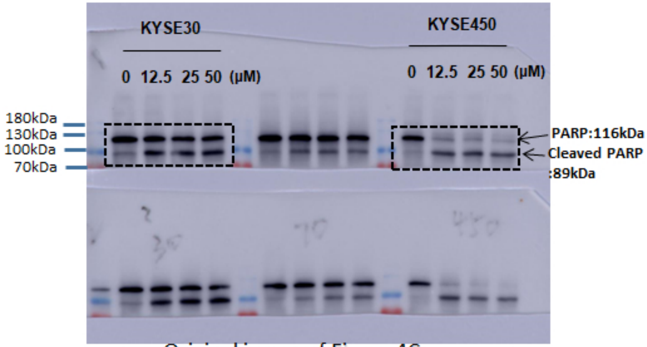

Original image of Figure 4C
with colorful protein ladder.

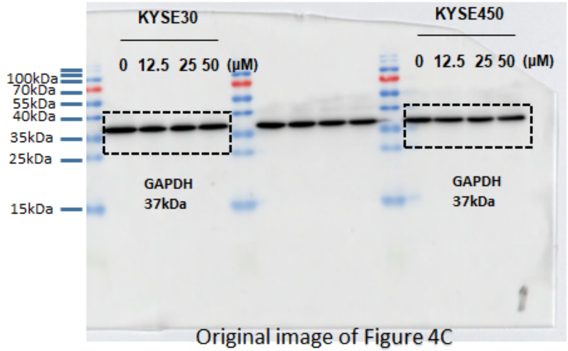

Original image of Figure 4C
with colorful protein ladder.

Figure S4

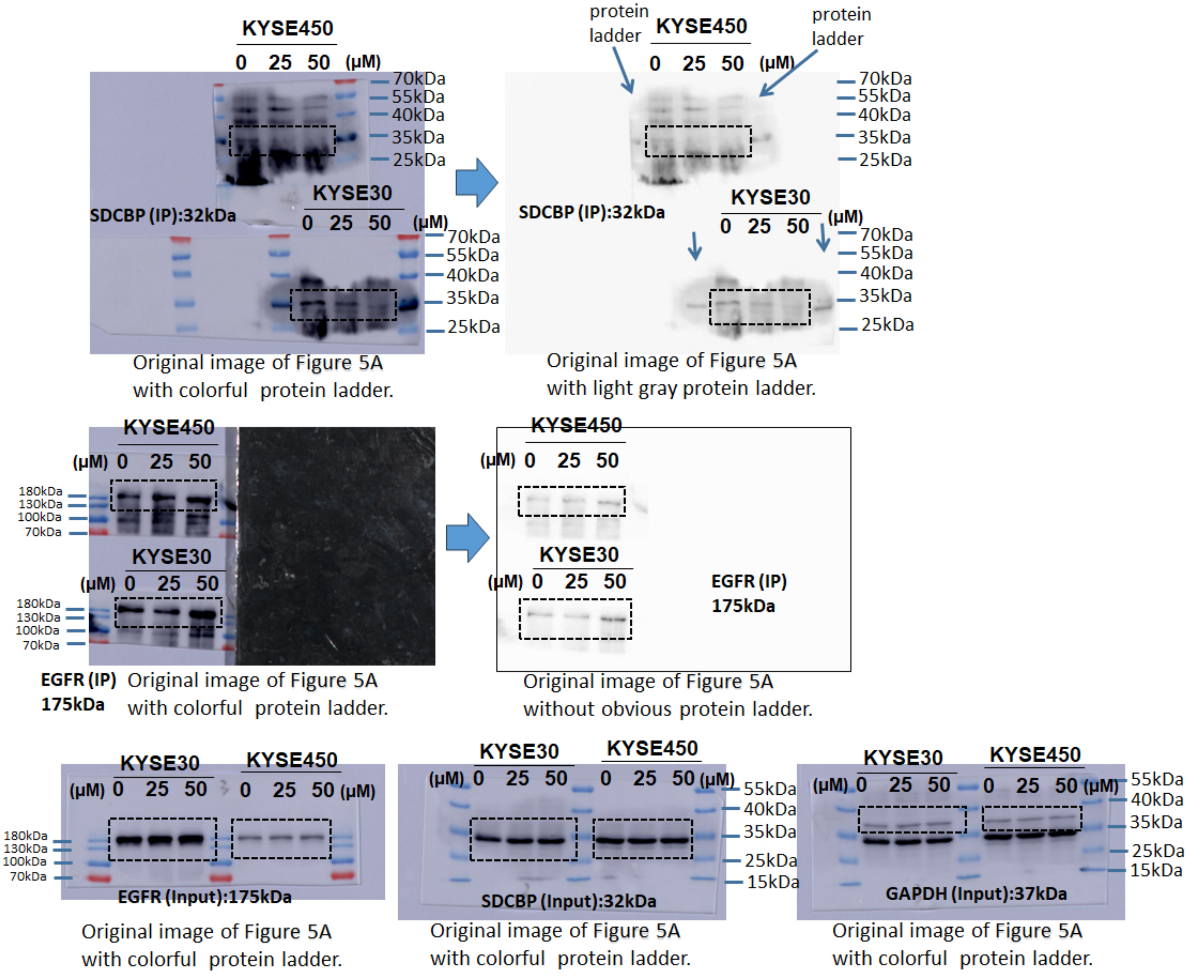
KYSE450
0 25 50 (μM)
70kDa
55kDa
40kDa
35kDa
25kDa
SDCBP (IP):32kDa
KYSE30
0 25 50 (μM)
70kDa
55kDa
40kDa
35kDa
25kDa
Original image of Figure 5A
with colorful protein ladder.
protein ladder
KYSE450
protein ladder
0 25 50 (μM)
70kDa
55kDa
40kDa
35kDa
25kDa
SDCBP (IP):32kDa
KYSE30
0 25 50 (μM)
70kDa
55kDa
40kDa
35kDa
25kDa
Original image of Figure 5A
with light gray protein ladder.
KYSE450
(μM) 0 25 50
180kDa
130kDa
100kDa
70kDa
KYSE30
(μM) 0 25 50
180kDa
130kDa
100kDa
70kDa
EGFR (IP)
175kDa
Original image of Figure 5A
with colorful protein ladder.
KYSE450
(μM) 0 25 50
KYSE30
(μM) 0 25 50
EGFR (IP)
175kDa
Original image of Figure 5A
without obvious protein ladder.
KYSE30
KYSE450
(μM) 0 25 50
0 25 50 (μM)
180kDa
130kDa
100kDa
70kDa
EGFR (Input):175kDa
Original image of Figure 5A
with colorful protein ladder.
KYSE30
KYSE450
(μM) 0 25 50
0 25 50 (μM)
55kDa
40kDa
35kDa
25kDa
15kDa
SDCBP (Input):32kDa
Original image of Figure 5A
with colorful protein ladder.
KYSE30
KYSE450
(μM) 0 25 50
0 25 50 (μM)
55kDa
40kDa
35kDa
25kDa
15kDa
GAPDH (Input):37kDa
Original image of Figure 5A
with colorful protein ladder.

Figure S5

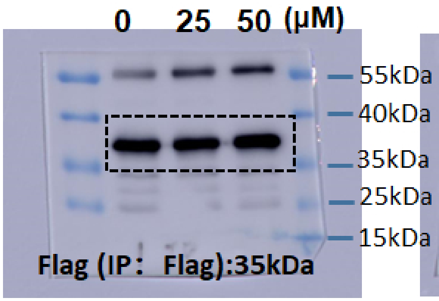

Original image of Figure 5B
with colorful protein ladder.

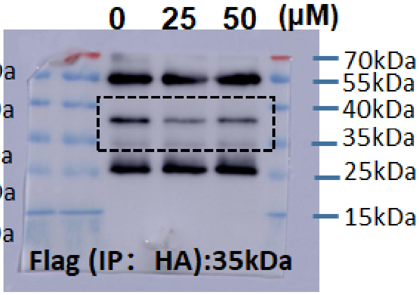

Original image of Figure 5B
with colorful protein ladder.

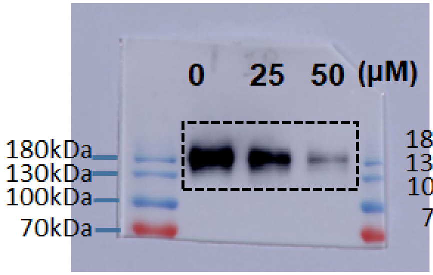

HA (IP： Flag): 176kDa
Original image of Figure 5B
with colorful protein ladder.

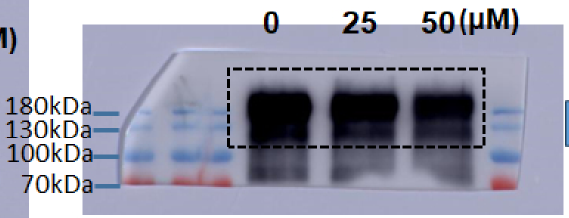

HA (IP： HA):176kDa

Original image of Figure 5B
with colorful protein ladder.

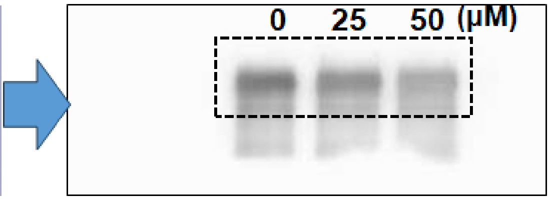

HA (IP： HA):176kDa
Original image of Figure 5B
without protein ladder.

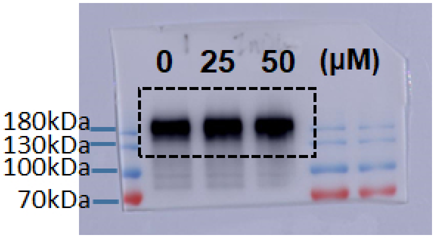

HA (Input):176kDa

Original image of Figure 5B
with colorful protein ladder.

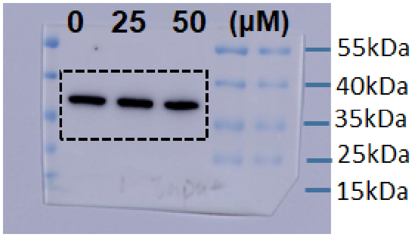

Flag (Input): 35kDa
Original image of Figure 5B
with colorful protein ladder.

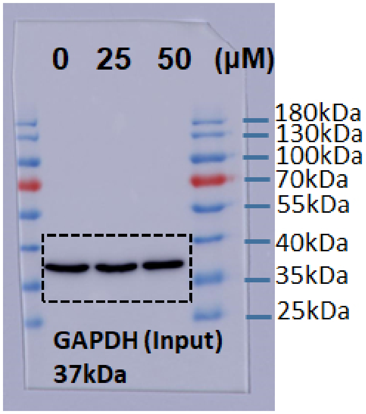

Original image of Figure 5B
with colorful protein ladder.

Figure S6

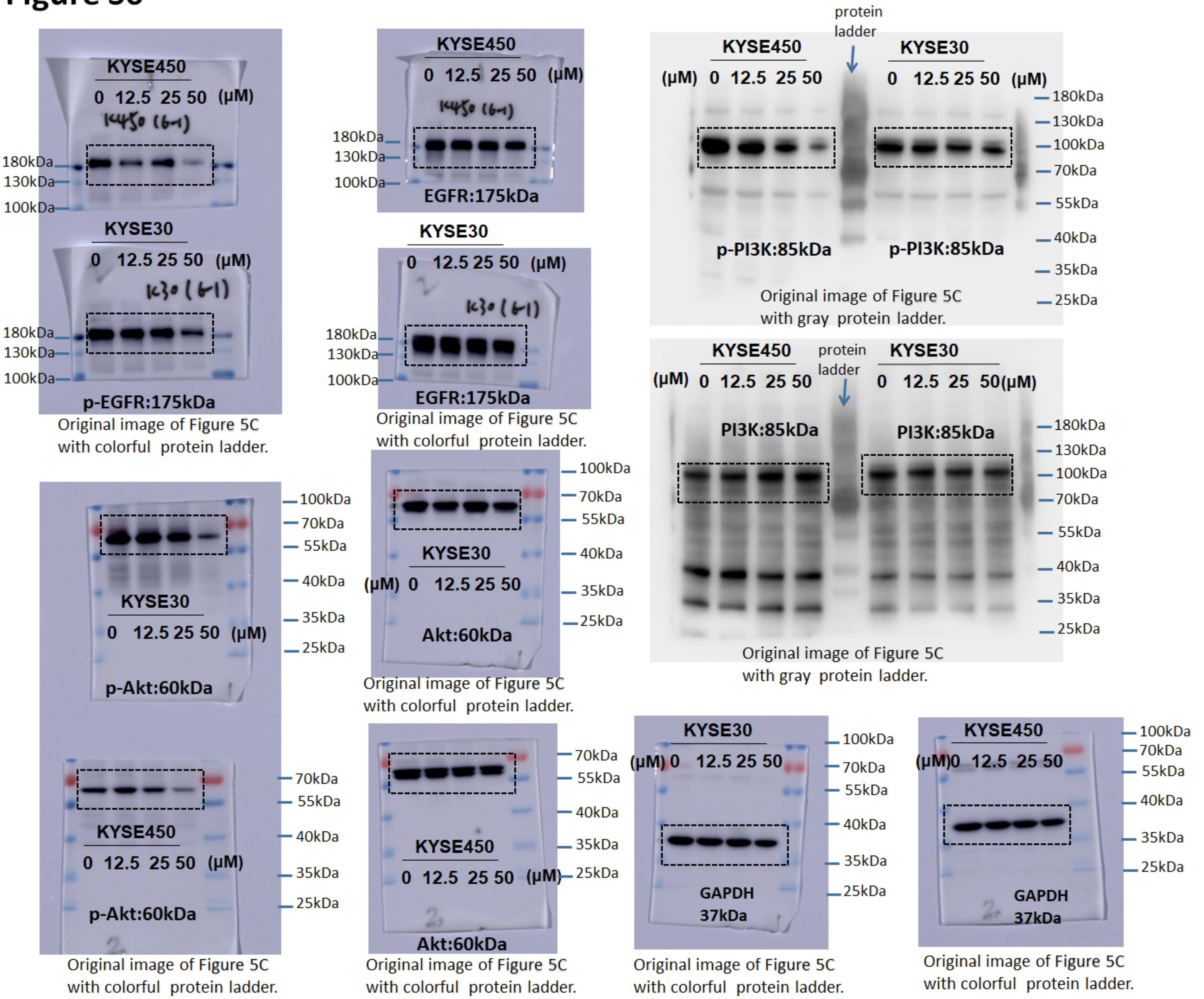
KYSE450
0 12.5 25 50 (μM)
180kDa
130kDa
100kDa
KYSE30
0 12.5 25 50 (μM)
180kDa
130kDa
100kDa
p-EGFR:175kDa
Original image of Figure 5C
with colorful protein ladder.
KYSE450
0 12.5 25 50 (μM)
180kDa
130kDa
100kDa
EGFR:175kDa
KYSE30
0 12.5 25 50 (μM)
180kDa
130kDa
100kDa
EGFR:175kDa
Original image of Figure 5C
with colorful protein ladder.
protein ladder
KYSE450
(μM) 0 12.5 25 50
KYSE30
0 12.5 25 50 (μM)
180kDa
130kDa
100kDa
70kDa
55kDa
40kDa
35kDa
25kDa
p-PI3K:85kDa
p-PI3K:85kDa
Original image of Figure 5C
with gray protein ladder.
KYSE450
protein ladder
KYSE30
(μM) 0 12.5 25 50
0 12.5 25 50(μM)
PI3K:85kDa
PI3K:85kDa
180kDa
130kDa
100kDa
70kDa
55kDa
40kDa
35kDa
25kDa
Original image of Figure 5C
with gray protein ladder.
100kDa
70kDa
55kDa
40kDa
KYSE30
0 12.5 25 50 (μM)
35kDa
25kDa
p-Akt:60kDa
70kDa
55kDa
40kDa
KYSE450
0 12.5 25 50 (μM)
35kDa
25kDa
p-Akt:60kDa
Original image of Figure 5C
with colorful protein ladder.
100kDa
70kDa
55kDa
40kDa
KYSE30
(μM) 0 12.5 25 50
35kDa
25kDa
Akt:60kDa
Original image of Figure 5C
with colorful protein ladder.
70kDa
55kDa
40kDa
KYSE450
35kDa
0 12.5 25 50 (μM)
25kDa
Akt:60kDa
Original image of Figure 5C
with colorful protein ladder.
KYSE30
(μM) 0 12.5 25 50
100kDa
70kDa
55kDa
40kDa
35kDa
25kDa
GAPDH
37kDa
Original image of Figure 5C
with colorful protein ladder.
KYSE450
(μM)0 12.5 25 50
100kDa
70kDa
55kDa
40kDa
35kDa
25kDa
GAPDH
37kDa
Original image of Figure 5C
with colorful protein ladder.
